# Shared neuropathological features of mild traumatic brain injury and anesthesia

**DOI:** 10.3389/fneur.2026.1852782

**Published:** 2026-08-07

**Authors:** Annabelle Shanks, Medha Pan, Elissa Letterio, Ramana Kolady, Jeffrey J. Bazarian

**Affiliations:** 1 Department of Emergency Medicine, University of Rochester School of Medicine and Dentistry, Rochester, NY, United States; 2 Department of Neurology, Yale School of Medicine, New Haven, CT, United States; 3 Center for Biotechnology Education, Johns Hopkins University, Baltimore, MD, United States; 4 Department of Sports Medicine and Nutrition, University of Pittsburgh, Pittsburgh, PA, United States

**Keywords:** anesthesia, blood-brain barrier, concussion, neuroinflammation, neuronal injury, perioperative, traumatic brain injury

## Abstract

Mild traumatic brain injuries are highly prevalent and can produce persistent sequelae, creating substantial personal and societal burdens. Recent evidence suggests that anesthesia administration for extracranial surgeries is both common and potentially problematic following concussive injuries. Despite this concern, few studies have assessed outcomes among concussion patients undergoing surgery, and clinical guidance for this population remains limited. This review examines shared neuropathological features between mild traumatic brain injury and anesthesia to identify potential mechanisms that could underlie poor outcomes following surgery in the setting of concussion. Both mild traumatic brain injury and anesthesia contribute to neuroinflammation and impaired blood-brain barrier function, resulting in neuronal and glial injury. When superimposed on the mechanical injury caused by concussion, these overlapping processes may contribute to secondary injury, delayed recovery, and worse long-term outcomes.

## 1 Introduction

Globally, over 27 million people suffered from traumatic brain injury (TBI) in 2016 (1) and half of the population is estimated to experience TBI at least once in their lifetime (2). Most TBIs are classified as mild traumatic brain injury (mTBI) or concussion, defined by brief loss of consciousness, amnesia, or confusion. The incidence of mild TBI has been rising, particularly among those under 5 and over 60 years of age. Primary mechanisms of injury include sports, falls, motor vehicle collisions, and assaults (3).

Mild TBI results from rapid deceleration and rotation of the head, causing neuronal axons to stretch beyond their physical limits, leading to chemical and cellular changes in the brain. This results in symptoms such as headaches, memory and attention problems, dizziness, insomnia, and mood disturbances (4, 5). While these symptoms typically resolve within 1 to 2 months, they can persist for more than 3 to 6 months in up to a third of patients, leading to school absenteeism, loss of employment, disrupted relationships, and reduced quality of life (6, 7). Symptom persistence is more common among females, those over 60, and individuals with a history of prior TBI, anxiety, or depression (8, 9). Identifying modifiable factors contributing to long-term disability is a public health priority. Here, we review evidence that anesthesia exposure after mTBI may represent a modifiable risk factor for prolonged concussion recovery.

Anesthesia may be administered after mild TBI to facilitate surgical repair of other injuries, such as bone fractures and internal organ injuries (10). The injured brain may be particularly vulnerable to anesthesia-associated physiologic and cellular stressors, potentially contributing to poor neurologic outcomes. During the early post-concussive period, the brain is highly susceptible to secondary injuries due to changes in cerebral blood flow, metabolism, and inflammatory response (10). Thus, the use of anesthesia for co-occurring injuries in patients with mTBI during this period may pose a risk to brain recovery. Despite the increasing burden of mild TBI worldwide, little is known about the frequency of anesthesia administered post-concussion. A 2017 study revealed that 20.6% of concussion patients received anesthesia within 1 year of injury, with a third of these incidents occurring within the first week post-injury (10). Although outcomes in that study were not reported, a recent study of 1,835 adult TBI patients (80% with mild TBI) from the Transforming Research and Clinical Knowledge in Traumatic Brain Injury (TRACK-TBI) research consortium indicated that anesthesia administered within 5 days of injury was associated with adverse functional outcomes and impaired executive function (11). To our knowledge, no other published studies have examined outcomes in mild TBI patients undergoing anesthesia, and there are no published recommendations or guidelines regarding the choice of anesthetic agent or timing of administration after mild TBI. In current medical practice, surgery and anesthesia often proceed without specific guidance accounting for recent mild brain injury.

**Figure 1:**
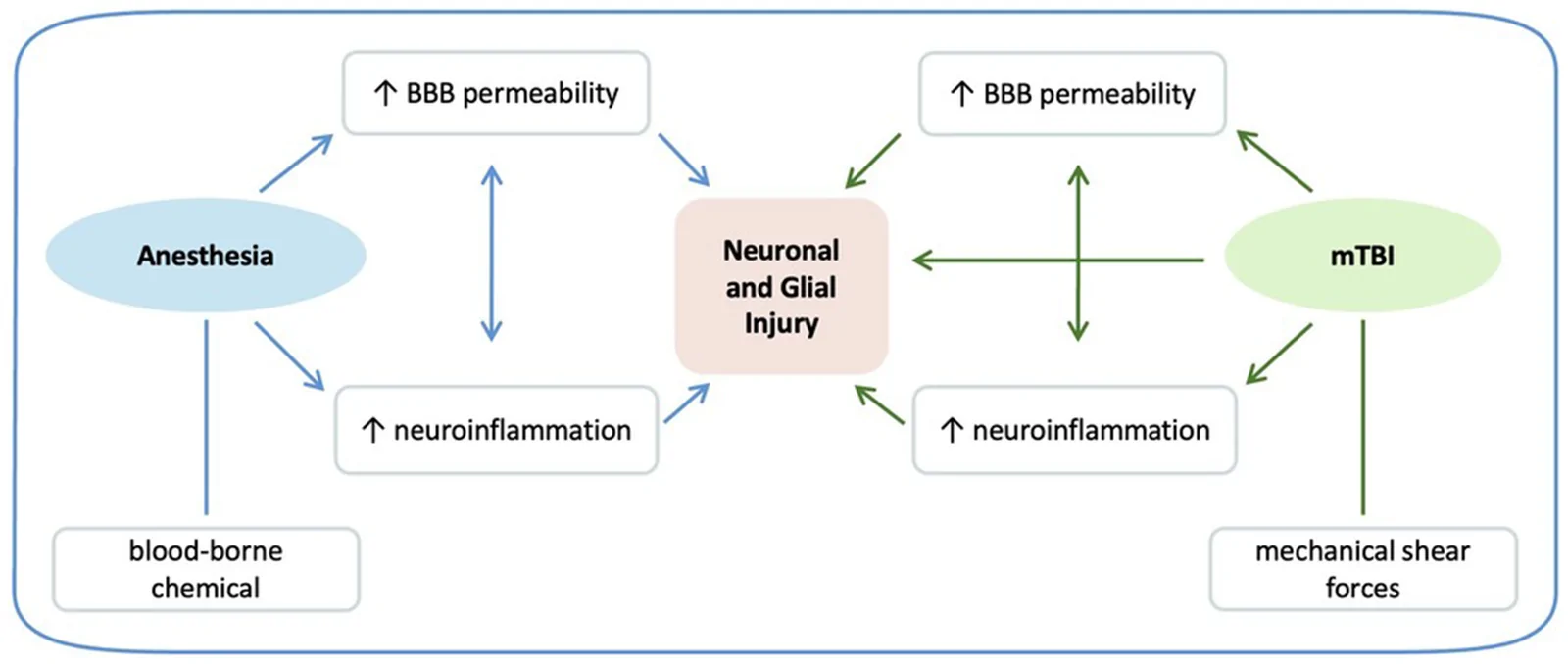
Anesthetic agents and mTBI may both increase BBB permeability and neuroinflammation, thereby contributing to neuronal and glial injury. In addition, mTBI-related shear forces can injure these cells directly. In both anesthesia- and mTBI-induced processes, increased BBB permeability both contributes to and is perpetuated by neuroinflammation.

Anesthesia and mild TBI both induce temporary changes in brain structure and function. While some cellular effects are unique to each condition, several overlap. These intersecting cellular effects have the potential to interact, leading to delayed recovery and greater secondary injury than either would alone. Given the paucity of clinical research on this topic, we sought to understand the potential dangers of combining anesthesia and mTBI by reviewing the neuropathologic effects they have in common: increased blood-brain barrier permeability, neuroinflammation, and neuronal and glial injury (Figure 1) (12–18). This review may provide a scientific basis for future clinical and translational studies aimed at defining the effects of anesthesia exposure in the days and weeks following mild TBI.

## 2 Increased blood-brain barrier permeability

### 2.1 BBB structure and function

The blood-brain barrier (BBB) consists of tight junctions (TJs) between adjacent endothelial cells lining blood vessels, the basement membrane, which separates neural tissue from blood circulation in the central nervous system, and astrocytic end-feet. Integrity of the barrier is crucial as it prevents harmful components circulating in the blood from entering the brain (19). Under normal conditions, only lipid-soluble molecules with a molecular weight less than 400 to 600 Da are able to diffuse across the BBB, unless a molecule-specific transmembrane carrier protein is present (20). Greater BBB disruption permits larger blood-derived components to enter the brain parenchyma (14).

Although BBB integrity depends on multiple structural components, tight junctions appear particularly vulnerable to both anesthesia and mTBI. TJs are formed by a small set of core proteins that physically seal adjacent endothelial cells and regulate barrier permeability. Disruption of these TJs compromises the integrity of the barrier, allowing potentially harmful molecules to cross via paracellular diffusion (12). Because TJs are sensitive to injury and anesthesia, subsequent sections will focus on mechanisms that alter key TJ proteins, zonula occludens-1 (ZO-1), occludin, and claudin-5. ZO-1 serves as a scaffolding protein, occludin contributes to junction stability, and claudin-5 is the primary determinant of BBB tightness.

Under normal conditions, BBB integrity is maintained by the Wnt/β-catenin signaling pathway, which causes downstream upregulation of ZO-1, occludin, and claudin-5 (21, 22). This signaling pathway is opposed by the NF-κB inflammatory signaling pathway, which promotes transcriptional upregulation of matrix metalloproteinase-9 (MMP-9), resulting in degradation of TJs and the extracellular matrix (23, 24). Both mTBI and volatile anesthetics may be involved in Wnt/β-catenin downregulation and NF-κB upregulation.

### 2.2 Evidence of increased BBB permeability following mTBI

Direct structural BBB disruption is commonly observed following mTBI, permitting normally separated blood components to enter brain tissue and contribute to secondary injury and edema (18, 25). Limbic and cortical areas are particularly vulnerable to injury-induced permeability changes (26, 27). Animal studies show size-dependent tracer leakage, such as Evans Blue, a marker of albumin extravasation (Table 1). Evans Blue leakage is evident from 1 to 7 days following mTBI, indicating increased paracellular leakage; however, interpretation is limited by the need for anesthetic exposure during injury induction (28). Furthermore, head rotation concussive injuries in swine are associated with multifocal extravasation of fibrinogen, a serum protein involved in blood clotting, into the parenchyma at 6–72 h post-injury (29). Serum protein extravasation was often colocalized with axonal pathology, suggesting potential toxic effects, and was rapidly taken up by surrounding neurons and astrocytes. In human studies, similar disruptions in BBB permeability have been observed. Gadolinium contrast (1 kDa) extravasation has been detected through dynamic contrast-enhanced MRI in professional mixed martial arts fighters and adolescent rugby players within 5 days of a fight or game (30). This early contrast leakage indicates acute BBB disruption and dysfunction of the neurovascular unit following sport-related impacts in humans.

**Table 1:** Summary of neuropathologic changes after mTBI, anesthesia, and both combined.

Pathophysiologic changes	Effect of mTBI	Effect of volatile anesthetics	Combined effects
BBB disruption			
A. Cellular effects			
Loss of TJ proteins	↑ NF-κB/MMP-9	↓ Wnt/β-catenin	↑ NF-κB/MMP-9
	↓ ZO-1, claudin-5, occludin	↓ ZO-1, claudin-5, occludin	↓ ZO-1, claudin-5
	↑ VEGF	↓ F-actin, VE-cadherin	
Changes in endothelial cells	None reported	Flattened luminal surfaces of capillary endothelial cells	Endothelial flattening
		↑ membrane fluidity	
B. Tissue effects			
Cerebral edema	↑ brain water content	↑ brain water content	↑ brain water content
	↓ CBF	↑ CBF	
	↑ ICP		
BBB leakage	Extravasation of cadaverine, Evans blue (albumin), gadolinium^*^ contrast	Extravasation of Evans blue (albumin), dextran	Extravasation of Evans blue (albumin), radioiodine
		Reduced TEER	
Neuroinflammation			
Shared cellular effects	↑ C3/C5 activation, ROS	↑ ROS, cyt C, caspase-3	↑ ROS, complement activity
Astrocyte activation	↑ A1 phenotype	↑ A1 phenotype	↑ GFAP
	↑ serum GFAP S100B^*^	↓ Ca^2+^ signaling	
	↑ TSPO PET		
Microglial activation	Chronic M1-like phenotype	↑ M1-like phenotype	↑ M1-like phenotype
	Acute M2-like phenotype		
Cytokine activation	↑ serum TNF, IL-6, and IL-1 family^*^	↑ serum TNF, IL-6, CRP, IL-10^*^	↑ serum TNF, IL-1 family
	↑ CSF IL-8^*^		
Neural/glial injury			
A. Cellular effects			
Neurons	↑ NADPH oxidase, ROS	↑ ROS, cytC, caspase-3, Aβ, p-tau	↓ NeuN, neurogranin, CaMKII
	↓ NeuN, parvalbumin, CaMKII – atypical neuron phenotype	↓ BDNF	
	↑ serum NfL, tau, UCH-L1, NSE^*^	↓ Bcl-2:Bax – ↑ apoptosis	
	↓ postsynaptic Homer1		
Astrocytes	↑ serum GFAP, S100B	↓ Ca^2+^ signaling	↑ serum GFAP
	↓ astrocytic Glt1, 1, S100B		↑ astrocytic GFAP
	↑ perinodal swelling		↑ activation and proliferation
Oligodendrocytes	↓ myelination, myelin lipids	↓ myelination	↓ myelination
		↑ apoptosis	
Microglia	↑ activation, MMP-9, TNF, prostaglandin E2, IL-1β	↑ activation and proliferation	↑ activation
	↓ NeuN	↑ TNF	↓ NeuN
	↑ NADPH oxidase, ROS		
B. Tissue Effects			
White matter integrity	Axonal swelling (↑ FA) and axonal loss (↓ FA) on DTI^*^	None reported	None reported

Aβ, amyloid-beta; Bax, Bcl-2 associated X protein; Bcl2, B-cell lymphoma 2; BDNF, brain-derived neurotrophic factor; CaMKII, Ca^2+^/calmodulin-dependent protein kinase II; CBF, cerebral blood flow; CRP, C-reactive protein; CSF, cerebrospinal fluid; cyt C, cytochrome C; C3/C5, complement components 3 and 5; DTI, diffusion tensor imaging; FA, fractional anisotropy; F-actin, filamentous actin; GFAP, glial fibrillary acidic protein; ICP, intracranial pressure; IL, interleukin; NeuN, neuronal nuclei; NF-κB/MMP-9, nuclear factor kappa B matrix metalloproteinase-9; NfL, neurofilament light chain; NSE, neuron-specific enolase; p-tau, phosphorylated tau; ROS, reactive oxygen species; S100B, S100 calcium-binding protein B; TEER, transendothelial electrical resistance; TJ, tight junction; TNF, tumor necrosis factor; TSPO, translocator protein; UCH-L1, ubiquitin C-terminal hydrolase L1; VE-cadherin, vascular endothelial cadherin; VEGF, vascular endothelial growth factor; ZO-1, zonula occludens-1. ^*^indicates evidence from studies in humans.

#### 2.2.1 Mechanisms of mTBI-Induced BBB dysfunction

In the setting of mTBI, BBB disruption occurs primarily due to mechanical shear stress and is later driven by resulting inflammation. These insults result in structural and functional TJ disruption. Regions with BBB leakage in mice subjected to mTBI correspond with decreased TJ expression for the key TJ proteins ZO-1, claudin-5, and occludin (15, 19, 31). ZO-1 disruption occurs within 10 min and lasts up to 24 h after injury, corresponding with the release of TJ protein-containing microvesicles following shear stress (15, 31, 32). This transcytosis of TJ proteins may underlie their reduced expression.

In addition to mechanical shear damage, mTBI triggers downstream inflammatory cascades that impact the BBB. Following mTBI, inflammatory signaling through the NF-κB pathway increases, which upregulates MMP-9 transcription and contributes to BBB dysfunction (19). Inflammatory activation of monocytes and astrocytes post-TBI triggers upregulation of vascular endothelial growth factor (VEGF), which degrades TJ proteins claudin-5 and occludin (19). Thus, synergistic mechanical and inflammatory pathways contribute to TJ-mediated BBB disruption following mTBI, forming a baseline of vulnerability that anesthesia may exacerbate.

### 2.3 Evidence of BBB permeability changes due to anesthesia exposure

Potentially compounding the increased BBB permeability from mTBI, exposure to certain anesthetic agents has also been shown to increase vulnerability to BBB disruption (16). Volatile anesthetics, such as sevoflurane (200 Da) and isoflurane (185 Da), are associated with impaired BBB function and extravasation of Evans Blue (961 Da) and dextran (10-kDa) into the parenchyma (16, 33). *In vitro* murine brain endothelial monolayer cell line studies also found that isoflurane and sevoflurane are associated with reduced transendothelial electrical resistance (TEER), a quantitative measure of barrier integrity, with lower TEER values indicating increased BBB permeability (31). These BBB changes may not consistently translate into meaningful edema in humans but may exacerbate injury-related edema.

Conversely, intravenous and intraperitoneal anesthetic agents, including fentanyl (336 Da), ketamine (238 Da), and pentobarbital (226 Da), appear to decrease or maintain BBB permeability (16). Rats anesthetized with either fentanyl or pentobarbital experienced reductions in extravasation of [14C] alpha-aminoisobutyric acid (103 Da) across the BBB (34). Propofol, an exception among intravenous agents, is associated with increased BBB permeability in both human and mouse *in vitro* studies as evidenced by reduced neurovascular endothelial cell resistance and greater sucrose uptake, respectively (35, 36). These agent-specific effects on BBB permeability highlight the need to define anesthetic strategies for patients with recent mTBI.

#### 2.3.1 Mechanisms of propofol- and volatile anesthesia-induced BBB disruption

Current evidence indicates three primary mechanisms by which volatile anesthetics, and to a lesser extent propofol, increase BBB permeability: downregulating TJ proteins, changing endothelial structure, and increasing endothelial membrane fluidity.

Exposure of rodents to 1.4% isoflurane for 2 h induced dextran extravasation, decreased TJ protein expression, and lowered β-catenin levels, consistent with downregulation of the Wnt/β-catenin signaling pathway responsible for maintaining TJ integrity (21, 22, 33). Unlike volatile anesthetics, propofol has not been shown to acutely decrease expression of key TJ proteins; however, it may contribute to post-acute TJ dysfunction as evidenced by reduced TEER and fluorescent microscopy (35–37).

Studies have also found decreases in filamentous-actin and vascular endothelial-cadherin, key structural components of the cytoskeleton, following anesthetic exposure (16, 33, 38). These proteins support cell-cell junctions, including TJs and adherens junctions, so their disruption may indicate both junction redistribution and cytoskeletal reorganization. The cytoskeletal changes result in flattening of luminal surfaces of endothelial cells, which is associated with increased BBB permeability (16, 22, 23, 39, 40).

Furthermore, due to their lipophilic properties, propofol, sevoflurane, and isoflurane readily incorporate into the lipid layers of endothelial cell membranes, making them more fluid. These changes can destabilize proteins that hold endothelial cells together, thereby compromising BBB integrity (36, 41, 42). Increases in membrane fluidity have been observed *in vitro* at dose levels that approximate those used clinically for propofol and volatile anesthetics, but not ketamine (42).

In addition to their direct effects on BBB integrity, volatile anesthetics may also disrupt the BBB indirectly through low-grade neuroinflammatory pathways. Mice exposed to 1.4% isoflurane who lack the gene for interleukin-6 (IL-6), a pro-inflammatory cytokine, have similar levels of BBB leakage to unanesthetized controls. On the other hand, mice able to express IL-6 demonstrate increased BBB permeability, suggesting that some level of inflammatory cytokine expression may be necessary for anesthesia-induced BBB disruption (33). MMP-9 has also been implicated in volatile anesthesia-associated BBB dysfunction, suggesting convergence with inflammatory pathways activated after mTBI (23).

### 2.4 Combined effects of mTBI and anesthesia on BBB permeability

Both mTBI and select anesthetic agents, particularly volatile anesthetics and propofol, can disrupt the BBB through two shared mechanisms (TJ disruption and neuroinflammation) and three distinct mechanisms: mechanical shear stress (mTBI), flattening of endothelial surfaces, and increased membrane fluidity (propofol and volatile agents). Despite common mechanisms and effects, there is limited research characterizing the combined effects of mTBI and anesthesia on BBB permeability.

While both volatile anesthetics and mTBI contribute to BBB breakdown, mTBI appears to have a greater effect than anesthesia alone. In mice exposed to either isoflurane or sevoflurane, those that underwent controlled cortical impact (CCI) had greater cerebral edema than control mice (31). CCI increased edema formation in an anesthetic-dependent manner, as the effect was greater after administration of isoflurane compared to sevoflurane (31). Sevoflurane may have a protective effect by increasing ZO-1 expression, reducing BBB disruption and subsequent brain water content, suggesting it may be preferable to isoflurane (31). As both agents are vasodilators, increased cerebral blood flow following their administration also contributes to cerebral edema, beyond BBB disruption. Without CCI, both anesthetics caused reduced TEER but were not associated with increased brain water content or reduced TJ expression 24 h after administration, suggesting these agents may cause small, transient deficits in BBB permeability. The combined effect of mTBI and anesthesia on TJ dysfunction may be partially driven by NF-κB/MMP-9 activation (43).

Although this relationship is difficult to isolate experimentally because TBI models often require anesthesia, available evidence suggests that select anesthetics may amplify BBB permeability after brain injury (31, 43).

## 3 Neuroinflammation

### 3.1 Key aspects of neuroinflammation

Beyond increasing BBB permeability, mTBI and anesthesia also trigger neuroinflammatory cascades that both depend on and further disrupt the BBB. The neuroinflammatory process involves complex signaling cascades, mediated by microglia and astrocytes, that evolve over the course of injury. The response often begins with activation of the complement cascade, followed by release of inflammatory chemokines and cytokines (e.g. interleukins-1,-6,-8 (IL-1,-6,-8), tumor necrosis factor (TNF), and C-reactive protein (CRP)), which promote immune cell recruitment and endogenous repair mechanisms (44–47). Many of these cytokines are released through NF-κB-dependent microglial signaling.

Microglia are key facilitators of the neuroinflammatory response. They typically exist in a ramified state but transition to an activated state in response to injury or infection (48). Activated microglia are categorized into M1-like and M2-like phenotypes and are characterized by increased Iba-1 expression. The M1-like phenotype produces high levels of cytokines, chemokines, and reactive oxygen species (ROS). This phenotype often emerges acutely and is downregulated once the insult has been neutralized. Chronic or excessive M1-like activation is associated with neurodegeneration (49). The M2-like phenotype is involved in phagocytosis, pro- and anti-inflammatory memory immune responses, and tissue remodeling (48). Under normal conditions, these activated phenotypes are suppressed by peroxisome proliferator-activated receptor-γ (PPARγ), which regulates inflammation by inhibiting pro-inflammatory pathways like NF-κB (45).

Like microglia, astrocytes undergo phenotypic changes in response to injury, adopting either an A1 or A2 phenotype. Inflammatory stimuli can cause astrocytes to adopt an A1 neurotoxic phenotype, leading to the release of complement proteins, chemokines, and cytokines (50). Conversely, astrocytes can also be induced to adopt an A2 neuroprotective phenotype, leading them to release anti-inflammatory cytokines, such as IL-10, and neurotrophic factors, such as brain-derived neurotrophic factor (BDNF) (50). Activated astrocytes are evidenced by increased levels of glial fibrillary acidic protein (GFAP) (51).

As mentioned in Section 1, these acute inflammatory events disrupt the BBB, leading to increased swelling and infiltration of blood contents into neural tissue (52, 53). Conversely, cytokines and chemokines may also support nerve growth factor (NGF) release, antioxidant responses, and neuroplasticity in the chronic stages of injury and disease (52, 54–57). These effects may promote neuron survival and adaptation, highlighting the context-dependent role of inflammation in recovery.

### 3.2 Glial inflammatory changes following mTBI

Neuroinflammation triggered by biomechanical damage is a major driver of secondary injury and cell death following mTBI (25), though many inflammatory processes also support recovery at later stages. Following moderate TBI and mild repetitive TBI, microglia have been shown to chronically adopt an M1-like phenotype, which perpetuates inflammation and impedes tissue repair (48). In mice that undergo CCI, microglia show an early shift toward an M2-like phenotype within the first week, followed by a transition toward chronic M1-like activation by 28 days post-injury (58). While the early M2-like response may facilitate tissue repair, chronic M1-like activation perpetuates inflammation and contributes to white matter injury (48).

Microglial activation can be quantified using [18F]DPA-714 micro-positron emission tomography (TSPO PET) where elevated TSPO signal reflects increased microglial activity (59). Higher TSPO signal is associated with greater injury severity and worse functional outcomes (59). Chronic TSPO signal elevation has been observed in former football players, indicating chronic neuroinflammation and microglial activation following repetitive head impacts (60). In animal models, this microglial activation is accompanied by neutrophil migration to injury sites for BBB repair, but no other overt cellular recruitment (61).

Astrocytes similarly shift to an A1 pro-inflammatory phenotype after injury, amplifying inflammatory signaling and reducing neuronal support (50). These glial changes, particularly after repetitive mTBI, are associated with reduced transcription of PPARγ (45). Increased ROS after injury due to oxidative stress may suppress PPARγ activity, perpetuating inflammation and contributing to deficits in spatial learning and memory (45, 62). Supporting its anti-inflammatory role, PPARγ overexpression reduces GFAP levels, indicating attenuation of chronic reactive gliosis (45, 51).

### 3.3 Neuroinflammatory signaling following mTBI

Neuroinflammatory signaling after mTBI involves complement proteins, chemokines, and cytokines, many of which exert both toxic and neuroprotective effects. Complement activation illustrates this duality: inhibiting the cascade with sCrry reduces BBB dysfunction and neurological impairment (63), while complement activator C3a promotes NGF release from microglia and may support recovery (53).

Similar dual patterns occur with cytokines. IL-8 remains elevated in the CSF for up to 21 days after head injury, resulting in both increased BBB dysfunction and NGF release (53). TNF impairs memory and motor function for the first 2 to 3 weeks after injury but shifts to neuroprotective effects by 4 weeks post-injury in mice (52). IL-6 likewise contributes to BBB leakage and ICP elevation, yet IL-6-deficiency results in greater oxidative stress and neural apoptosis after injury, suggesting a role in long-term recovery (54, 64).

S100B, a calcium binding protein released by astrocytes and other cell types, may also be involved in the post-injury inflammatory response (65). At low extracellular levels, it serves to prevent apoptosis and stimulate astrocytes, while at higher levels it causes neuronal death and neuroinflammation via stimulation of microglia and astrocytes (57). Serum S100B elevations have been reported in mixed martial arts athletes after fights (30) and have been linked to unfavorable outcomes in TBI patients (66).

### 3.4 Detrimental neuroinflammatory effects of anesthesia

Pharmacologic CNS depression and postoperative systemic inflammation can result in mild inflammatory activation after anesthesia, particularly in aged or vulnerable brains. Prolonged exposure is particularly problematic, leading to microglial activation and suppression of neural activity, ultimately resulting in perioperative neurocognitive disorders (67). Anesthesia-associated neuroinflammation is particularly relevant in older adults, in whom it may contribute to postoperative delirium (33, 68) and in perinatal infants due to effects on neurodevelopment (69).

Sevoflurane and isoflurane are associated with increased BBB permeability and elevated levels of pro-inflammatory cytokines, including IL-6, TNF, and CRP, through NF-κB activation (24, 40, 67, 70–72). This activation leads to initiation of the complement cascade and the promotion of a pro-inflammatory M1 microglial phenotype (24). Propofol may contribute to neuroinflammation by stimulating endothelial cells within the brain and by triggering stress pathways and inflammatory signaling, ultimately resulting in BBB disruption (17). A rise in IL-10, an anti-inflammatory cytokine, following anesthesia may reflect a compensatory anti-inflammatory response (17).

### 3.5 Neutral and neuroprotective effects of anesthesia

Although several anesthetic agents aggravate an inflammatory response, others appear neutral or protective. Desflurane, for instance, does not affect levels of IL-6 in mice, suggesting minimal inflammatory involvement (70).

Anesthetics within the α2-agonist class exhibit neuroprotective anti-inflammatory effects, which may offset neuroinflammation caused by other anesthetics or TBI (22, 73). Dexmedetomidine, a highly selective α2-agonist, decreases both neuroinflammation and ICP by inhibiting NF-κB-mediated cytokine production and reducing cerebral blood flow (22, 74). Together, these effects may protect aged mice from anesthesia-induced cognitive impairment.

### 3.6 Combined effects of mTBI and anesthesia on neuroinflammation

Both mTBI and volatile anesthetics promote neuroinflammation, and these effects are further exacerbated by BBB dysfunction (24, 25, 48, 53, 59, 63, 70–72). Together, these insults may trigger microglial activation and the initiation of the inflammatory cascade, leading to the release of inflammatory cytokines and chemokines, which damage tissue and increase the risk of adverse outcomes (48, 54, 58, 63, 64, 67, 70–72). As both mTBI and volatile agents independently contribute to these neuroinflammatory processes, their combined effects likely amplify overall inflammation.

However, some anesthetic agents, including dexmedetomidine and propofol, may counteract aspects of the mTBI-induced inflammatory response. Propofol, despite debate surrounding its pro- vs. anti-inflammatory effects, attenuated post-TBI microglial activation and neuronal loss, likely through reduced expression of TNF, IL-1β, and ROS (75). This contrasts with volatile anesthetics, which more often appear to amplify neuroinflammatory cascades.

## 4 Neuronal and glial injury

### 4.1 Key aspects of neuronal and glial injury

Neuronal and glial injury encompasses structural and cellular damage that can impair processes, such as neuronal plasticity, neurogenesis, and axonal transport. The brain attempts to adjust to these insults through neuronal plasticity, its capacity to reorganize synaptic and structural connections. Plasticity and neurogenesis are involved in basic neuronal processes, such as learning and memory, and are crucial to the brain's ability to compensate for injury. Anesthesia-induced deficits in these processes may hinder recovery following mTBI.

Following TBI, cell death may occur through multiple overlapping pathways, involving apoptosis, necrosis, excitotoxicity, calcium influx, mitochondrial dysfunction, oxidative stress, and inflammatory signaling (5). Typically, enzymes important for apoptosis, such as caspase-3, are activated by the calcium ion influx induced by the injury (76). In mild TBI, overt neuronal loss may be limited, but sublethal neuronal dysfunction, atypical neuronal phenotypes, synaptic instability, and axonal injury can still impair recovery (14, 77). In more severe injury, these same pathways may progress to more extensive neuronal and glial cell death. Anesthesia exposure during a period of post-injury vulnerability may plausibly exacerbate these processes by altering mitochondrial function, inflammatory signaling, cerebral perfusion, and apoptotic pathway activation (18, 78, 79).

### 4.2 mTBI-induced neuronal injury

mTBI can cause neuronal injury both directly, through shear stress, and indirectly, via cerebral microbleeds, BBB leakage, and neuroinflammation (Figure 1). Traumatic axonal injury (TAI) results from the mechanical shear stress of mTBI. TAI involves stretching, twisting, and tearing of axons due to rotational and linear acceleration, resulting in impaired axonal transport and demyelination (77). This process manifests as multifocal decreases in fractional anisotropy (FA) on MRI diffusion tensor imaging, indicating reduced white matter integrity (80). Oligodendrocytes may eventually remyelinate the damaged axons, but the resulting myelin sheaths are reduced in thickness compared to axons with similar diameters, possibly owing to selective loss of myelin lipids (80, 81). Although oligodendrocytes may not die after mTBI, mature and actively myelinating oligodendrocytes may lose their differentiated phenotype in response to injury (81).

Shear stress also results in cerebral microbleeds (CMBs), which allow plasma proteins to enter the parenchyma, causing abnormal neuronal phenotypes that represent a secondary form of neural injury. CMBs are associated with sustained loss of neuronal and calcium activity markers within minutes of mTBI in mice (14, 82, 83). Importantly, these decreases do not reflect apoptosis, but rather active, coordinated degradation of proteins, producing an “atypical” neuron phenotype (14). Atypical neurons may persist for years after injury, causing long-term deficits. Cumulative injuries appear to be more damaging; for example, two to three concussive events have been associated with mild neuronal loss in pigs (84).

Neuronal injury following mTBI is also evidenced by instability of immature synapses and a reduction in synaptic plasticity. Presynaptic terminals appear to be unaffected; however, immature postsynaptic terminals experience significant instability (14). Compared with sham animals, mice with mTBI showed unchanged synaptic density and dendritic spine length on Golgi staining, yet wider spine heads. This indicates a shift toward stronger, more stable synapses, demonstrating decreased synaptic plasticity in cortical pyramidal cells (14). Mice subjected to mTBI showed increased mushroom, filopodia, and branched spines and decreased thin spines compared to controls (14). As thin spines are associated with learning processes, their decrease may be associated with reduced neuroplasticity after mTBI or could indicate the disruption of immature connections (85). Increases in filopodia may reflect attempts to restore plasticity, but whether this response is effective remains unclear (14). Overall, preliminary findings suggest that postsynaptic structures become less plastic following mTBI, which may contribute to persistent cognitive symptoms.

These functional deficits likely stem, in part, from the excitotoxic cascade that follows mTBI. Biomechanical injury results in mechanoporation of neuron membranes, allowing ion flux that ultimately depolarizes neurons, leading to indiscriminate glutamate release (5). As neurons attempt to restore their ion gradients, a high energy demand develops that is uncoupled with normal to low cerebral blood flow (5, 30). This mismatch between high energy demand and limited energy supply forces mitochondria to sequester accumulating calcium ions from glutamate signaling, resulting in mitochondrial dysfunction and oxidative crisis (5). In severe cases, this bioenergetic failure can contribute to necrotic cell death after TBI.

### 4.3 mTBI-induced glial injury

Glia are also affected by mTBI, primarily through secondary injury mechanisms. For example, entry of blood contents into the brain after cerebral microbleeds and BBB disruption may exert toxic effects on neuroglia. Regions around vessels that have BBB leakage following TBI in mice contain abnormal astrocytes, characterized by reduced expression of astrocytic proteins, including glutamate transporter-1 (Glt1) and S100B (14, 15). Rather than cell loss, these changes reflect a shift toward an atypical astrocyte phenotype, as cell density remains unchanged (14). This astrocyte dysfunction impairs BBB restoration, leading to sustained barrier disruption and prolonged exposure to blood products (15, 71).

ROS, such as ${\text{O}}_{2}^{-}$, also play a major role in glial injury following mTBI due to mitochondrial damage and dysfunction. ROS production from astrocytes, microglia, and neurons occurs in two temporal peaks: an early surge at 1 h post-injury and a delayed surge between 24 and 96 h due to mitochondrial activation (86). These ROS surges amplify glial cytokine production and further exacerbate BBB disruption, creating a self-reinforcing injury loop (19, 87). The hippocampal CA1 region and cerebral cortex are particularly vulnerable to ROS damage, as they experience increased NADPH oxidase activity, which produces ROS after TBI (86).

### 4.4 Effect of anesthesia on neuronal and glial injury

Unlike mTBI, anesthesia does not directly impose mechanical injury, but it may contribute to neuronal and glial injury through indirect cellular pathways (Figure 1). Anesthesia-associated inflammatory and neurotoxic signaling can promote apoptosis and impair synaptic plasticity, particularly in young or otherwise vulnerable brains (78, 79).

#### 4.4.1 Mechanisms of anesthesia-induced injury

Anesthesia-induced neuroapoptosis in young or vulnerable brains may impair plasticity and neuronal communication, although these effects appear less common in adults. Early postnatal rodent studies suggest that general anesthesia exposure can induce cognitive and behavioral deficits associated with neurotoxicity and apoptosis. Isoflurane activates both extrinsic and intrinsic apoptotic pathways. Extrinsically, inflammation-mediated activation of TNF receptors promotes apoptosis, while intrinsic pathways are driven by mitochondrial release of pro-apoptotic factors (78). Isoflurane shifts the balance toward pro-apoptotic signaling, increasing cytochrome c and caspase-3 cleavage, which are hallmarks of intrinsic apoptosis (79).

Isoflurane exposure is also associated with increases in ROS (79). Oxidative stress may serve as a convergent pathway through which mTBI and anesthesia amplify neural injury. By promoting mitochondrial dysfunction, inflammatory signaling, BBB disruption, and apoptotic pathway activation, ROS may contribute to the secondary injury cascade when anesthesia exposure occurs during the post-TBI vulnerability window.

Structural integrity may also be vulnerable to anesthesia exposure. Propofol, midazolam, and volatile anesthetics can inhibit BDNF signaling, which has been associated in rodents with reduced dendritic spine expression and impaired axonal transport related to microtubule loss (78).

Glial cells are also vulnerable to anesthesia exposure. Chronic general anesthesia exposure, especially during developmental periods, increases microglial activation. Propofol and sevoflurane have been associated with increased microglial quantity and activation in the human fetal prefrontal cortex (18). Anesthetic-induced oligodendrocyte apoptosis has been linked to reduced axonal myelination and cytoskeletal damage, further compromising white matter integrity (78). While neurons may become more robust to anesthetic exposure as they age, oligodendrocytes remain vulnerable for a longer period. Juvenile rhesus macaques exposed to isoflurane had over 3 times more apoptotic cells than controls, with two thirds of these cells being oligodendrocytes (88). This anesthesia-induced reduction in oligodendrocytes could exacerbate existing axonal injury from mTBI.

### 4.5 Combined effects of mTBI and anesthesia on neuronal and glial injury

While only mTBI can directly cause neural injury through mechanical stress, both anesthesia and mTBI can lead to secondary injury through BBB disruption and neuroinflammatory processes that allow exposure to neurotoxins (71, 86). These downstream events result in atypical neuronal and astrocytic phenotypes, reduced plasticity, and, in some cases, cell death (14, 15, 78, 89). Together, these overlapping pathways may exacerbate neuronal and glial injury after mTBI and may contribute to clinically observable disturbances in cognition and arousal, including delirium.

### 4.6 Post-traumatic and postoperative delirium

The combined effects of neurotrauma and anesthesia on BBB disruption, neuroinflammation, and neural injury may increase vulnerability to delirium, an acute disturbance in attention, awareness, and cognition that is common after both TBI and surgery. Up to two thirds of patients who survive TBI develop agitation or delirium, and approximately 30% of older surgical patients experience postoperative delirium (90, 91). Among patients with mild to moderate TBI, nearly half develop delirium within the first 4 days after injury (92). Brain injury and surgery therefore represent important precipitating risk factors for delirium in critically ill patients (93).

Although the pathogenesis of delirium remains incompletely understood, proposed mechanisms include neuroinflammation, neurotransmitter imbalance, and neural injury; these pathways overlap with mechanisms implicated in both TBI and anesthesia-associated neurotoxicity (90, 93). Serum biomarkers associated with central nervous system injury and neuroinflammation, including S100B and IL-6, have been linked to delirium, suggesting that both structural injury and inflammatory cascades may contribute to acute post-traumatic cognitive dysfunction (93). In critically ill patients, dexmedetomidine may be protective against delirium relative to other sedative strategies, potentially owing to its anti-inflammatory and intracranial pressure-modulating effects (90).

Postoperative delirium is of particular concern in older adults, as advanced age increases susceptibility to anesthetic-associated BBB compromise, which may contribute to disrupted neural function (33, 40). These perioperative disturbances may also have implications beyond the immediate recovery period, as delirium has been associated with longer-term cognitive decline in older patients. In this context, propofol may represent a comparatively favorable anesthetic option for mitigating postoperative neurocognitive dysfunction, although further studies are needed to determine whether specific anesthetic strategies reduce delirium risk in patients recovering from TBI (68).

## 5 Discussion

Both mTBI and volatile anesthetics are associated with reduced BBB integrity, neuroinflammation, and neuronal and glial injury (14–16, 30, 31, 33, 94). Volatile anesthetic agents may transiently increase BBB susceptibility to disruption, potentially exacerbating BBB breakdown from biomechanical mTBI and overlapping neuroinflammatory pathways (15, 16, 22, 25, 31, 33, 52–54, 57, 67, 95). The combination of BBB dysfunction, neuroinflammation, and primary injury mechanisms may contribute to both direct neuronal damage and prolonged glial activation, particularly in young and aged brains (14, 78, 96–99). Thus, although their relative contributions may differ, both anesthesia and mTBI can promote BBB dysfunction, neuroinflammation, and neural injury. These processes may reinforce one another, amplifying secondary injury; however, research examining their combined effects remains scarce.

Despite their shared pathologic features, to our knowledge, only one study has directly examined the association between anesthesia exposure and recovery after mTBI. Roberts et al. conducted a retrospective analysis to assess clinical outcomes after extracranial surgery in patients with a wide range of TBI severity (11). At 2 weeks and 6 months after EC surgery, mTBI patients (GCS ≥13) who had positive findings on CT scan (complicated mTBI) had lower Glasgow Outcome Scale–Extended for all injuries (GOSE-ALL) scores, lower GOSE-TBI scores, and a 10% greater prevalence of cognitive impairment after extracranial surgery compared to their non-surgical counterparts (11). Conversely, mTBI patients who had negative findings on CT scan (uncomplicated mTBI) and orthopedic trauma controls (no mTBI) had similar outcomes regardless of whether they underwent surgery.

Differences between complicated and uncomplicated mTBI patients exposed to anesthesia suggest that the extent of BBB disruption may influence recovery after extracranial surgery. Further research is needed to characterize the combined effects of mTBI and anesthesia. Future animal and human studies should account for anesthetic agent, timing of exposure, and duration of exposure. Additional studies should address gaps in human evidence, including the extent of BBB leakage in patients with mTBI after anesthesia administration and the effects on neuronal and astrocytic markers.

Roberts et al. also found that the association between extracranial surgery and worse recovery extended to patients with moderate and severe TBI. Sedative and anesthetic management during the acute stages of injury may be beneficial in these patients for ICP control and seizure prevention (100); however, anesthesia exposure may be associated with worse outcomes. Similar to patients with complicated mTBI, patients with moderate and severe TBI who underwent extracranial surgery had significantly worse functional outcomes than their non-surgical counterparts (11). This association may reflect more pronounced activation of overlapping mechanisms implicated in mTBI and anesthesia, including BBB disruption, neuroinflammation, oxidative stress, and neural injury, but may also stem from hemodynamic instability and reductions in cerebral perfusion in the context of anesthesia (101).

Given the potential risk of anesthesia exposure after concussion, and the broader concern that anesthesia may exacerbate secondary injury across the TBI spectrum, recommendations regarding the timing of elective surgery after head injury may be warranted. However, establishing such guidelines will require a clearer understanding of whether there is a specific post-injury window during which the concussed brain is particularly vulnerable to anesthesia-associated secondary injury. To assess the effects of extracranial surgery timing on TBI recovery, Zheng et al. compared 6-month outcomes in TBI patients who underwent early fixation of orthopedic injuries, defined as within 24 h of injury, with those who underwent later fixation outside this window (102). The investigators found no significant difference in functional outcomes by surgery timing, suggesting that fixation within vs. after 24 h may not meaningfully alter recovery. However, this study did not consider the effects of complicated vs. uncomplicated mTBI and the possibility that adverse interactions with the surgical environment may persist beyond the 24-h post-injury window. Future studies could build upon this work by assessing whether outcomes vary across different post-injury time windows and between patients with varying CT findings.

The distinction between complicated and uncomplicated mTBI also highlights the need for more clinically relevant brain injury classifications. Without surgery, mTBI patients with positive findings on CT scan have a significantly worse 1-year prognosis than those with uncomplicated mTBI (103). Thus, CT findings may be critical to determining the effects of anesthesia on patient outcomes. Biomarkers may also play a role in identifying patients with complicated mTBI, as they tend to have marked increases in serum GFAP and neurofilament light (NFL) (56). Patients with complicated mTBI may require additional TBI education, closer follow-up, and potentially more conservative anesthetic planning. These groups may represent clinically distinct phenotypes and warrant separate classification in future studies.

## 6 Conclusion

Although separate lines of research suggest that anesthesia and brain injury share pathological features that may contribute to poor recovery and adverse long-term outcomes, their combined effects remain poorly understood. Studies examining both anesthesia exposure and brain injury are limited by important methodological constraints. Animal studies are limited by the ethical and practical challenges of inducing brain injury without anesthesia, creating an unavoidable confounding variable. Future studies assessing recovery outcomes alongside markers of BBB permeability, neuroinflammation, and neurotoxicity after mTBI and anesthesia exposure may help address these gaps.
